# Stakeholder perspectives on machine learning models predicting diabetic foot ulcers and amputations in diabetes care: a scenario-based interview study

**DOI:** 10.3389/fdgth.2026.1773356

**Published:** 2026-08-21

**Authors:** Iris ten Klooster, Saskia M. Kelders, Hanneke Kip, Rik Crutzen, Lisette van Gemert-Pijnen

**Affiliations:** 1 Centre for eHealth and Wellbeing Research, Department of Psychology, Health and Technology, University of Twente, Enschede, Netherlands; 2 Optentia Research Unit, North-West University, Vaal Triangle Campus, South Africa; 3 Department of Research, Stichting Transfore, Deventer, Netherlands; 4 Department of Health Promotion, Care and Public Health Research Institute, Faculty of Health, Medicine and Life Sciences, Maastricht University, Maastricht, Netherlands

**Keywords:** diabetes, healthcare, interview study, machine learning, prediction models, value proposition

## Abstract

**Background:**

Predictive machine learning models can support timely interventions for diabetes management. However, there is limited insight into stakeholder perspectives on their use in healthcare, which is important for alignment with expectations and in turn fostering adoption.

**Objective:**

This study aimed to identify stakeholder perspectives on the (1) scenarios of use, and (2) the values and attributes of two machine learning models predicting diabetic foot ulcers and amputations in diabetes care.

**Materials and Methods:**

Five diabetes patients, two healthcare workers and four experts involved in system integration aspects participated in semi-structured interviews. Three scenario components were presented to help participants articulate their needs and preferences. Attributes were inductively identified from interview data and directly linked to scenarios of use, with values analyzed based on the identified attributes.

**Results:**

We identified four scenarios of use namely (1) supporting healthcare workers’ decision making, (2) supporting patient empowerment, (3) improving appointment planning based on risk, and (4) providing early warnings concerning acute risks. In addition, thirteen values were identified: supporting patient awareness, hybrid approach, detecting acute risks, collaboration between healthcare workers at different levels, unobtrusiveness, integration into existing systems, explainability, translating patient data from EHRS into clinically relevant insights, risk-based stratification of care, continuous development, interoperability, privacy, and regulatory compliance.

**Discussion:**

The insights of this study can guide the creation of a system that integrate machine learning models for the prediction of diabetic foot ulcers and amputations.

## 1 Introduction

Diabetes mellitus (DM) is one of the most common chronic diseases, and the prevalence of DM is expected to continue to increase (1, 2). Globally, between 5.4 and 7.3% of diabetes patients have diabetic foot ulcers (DFU) (3). DFUs pose a significant burden on healthcare systems and have a negative impact on diabetic patients' quality of life, especially those leading to amputations (4, 5). Self-management plays an important role in the prevention of DFUs, with regular foot inspections being an important aspect (6, 7). Yet, previous studies show that many patients do not consistently perform regular foot inspections (8), particularly patients who perceive a low risk for developing DFUs (9, 10). Moreover, self-management can be difficult for patients with DM due to various obstacles. These include poor vision and visibility (10–12) and reduced foot sensitivity (13). These limitations hinder their ability to detect early symptoms of DFUs, resulting in unrecognized symptoms and, consequently, a lack of timely signals to engage in self-care activities, such wearing appropriate footwear or protecting feet from extreme temperatures (14, 15). Addressing these barriers requires novel methods and monitoring techniques to enhance early detection and support patients in managing their health effectively rather than on disease management (16).

An example of a method for early detection is the use of machine learning (ML) to predict future risk of developing DFUs and amputations. ML is a subset of artificial intelligence (AI) that focuses on developing algorithms that allow computers to learn from and make predictions based on historical patient data (17). These algorithms work by detecting patterns in patient data, where a portion of the dataset is used for training the model to recognize risk factors (the training set), and the remaining portion is reserved to test and validate the model's performance, through measures such as accuracy and precision (the test set). ML models have potential to predict various diabetes-related complications, including microvascular issues using variables like gender, age, and body mass index (18), and the recurrence of foot ulcers in high-risk patients (19). A promising data source for developing these models are electronic health records (EHRs), which provide a rich repository of longitudinal data, including a patient's medical history, diagnoses, medications, treatment plans, and laboratory results. The primary role of EHRs is to offer healthcare professionals accurate, up-to-date information to ensure coordinated care, but increasingly, they are being leveraged for the development of prediction models to enhance patient outcomes (20). The development of a ML model to predict DFUs and amputations based on EHR data might, for example, allow for targeted and timely interventions, such as foot care education to improve self-management behaviors in diabetes patients, or to create patients' awareness of the risk of developing foot ulcers motivating them to take preventive actions (21).

While numerous ML models demonstrate sufficient performance and offer promising solutions to various healthcare challenges, such as limited access to high-quality care, medical errors, and workforce shortages (22, 23), its widespread adoption in healthcare settings remains low (24, 25). As a result, ML applications often remain limited to pilot projects or academic research, with a significant gap between their potential and actual integration into routine clinical practice (24). This gap in widespread adoption in healthcare stems from several factors, such as difficult integration into EHRs, the need for educating clinicians about the use of AI, and integration within existing workflows (23, 26). Furthermore, previous studies suggest that performance metrics alone do not reflect clinical applicability (24), highlighting the need for research that examines how ML technologies can better align with the needs and expectations of end-users and other stakeholders in diabetes care to accommodate diverse perspectives (27). The Unified Theory of Acceptance and Use of Technology (UTAUT) provides a structured way to understand and address the needs and expectations of stakeholders when implementing ML technologies in diabetes care. While performance metrics focus on how well a machine learning model works, the UTAUT model emphasizes broader factors that influence adoption, such as performance expectancy, effort expectancy, social influence, and facilitating conditions (28).

To address the gap between potential and practical adoption, it is important to explore not only how ML technologies perform but also how they are envisioned to be used in real-world healthcare settings according to stakeholders, e.g., personalizing patient treatment plans or identifying risk factors for each patient (29). One way to achieve this is by identifying and describing scenarios of use, which capture detailed narratives of how the ML model might interact with people (e.g., patients, clinicians), activities (e.g., clinical workflows, decision-making), contexts (e.g., hospital environments, home care), and technologies (e.g., EHR systems, monitoring devices) (30). These scenarios, structured by the PACT framework, provide a comprehensive understanding of the practical and contextual factors influencing ML implementation. In addition to scenarios, examining the values and attributes associated with ML technologies helps ensure their design aligns with the needs and priorities of stakeholders. Values represent overarching benefits, such as explainability, privacy, or patient empowerment, that the technology provides to its stakeholders (31). Attributes describe specific characteristics or features of the system that incorporates the ML model that operationalize these values (32). Together, scenarios, values, and attributes provide a structured approach to designing ML technologies that are both clinically relevant and aligned with stakeholder expectations, thereby increasing their likelihood of adoption and meaningful use in healthcare practice.

This study is part of a larger study in which two predictive machine learning models are developed for the prediction of diabetic foot ulcers and amputations using data from EHRs. In the current study, we aim to identify stakeholders' views on the (1) scenarios of use of two machine learning models predicting DFU and amputations in diabetes care, (2) the values and attributes two machine learning models predicting DFUs and amputations.

## 2 Materials and methods

We used a qualitative research method to gain insight into stakeholders' views on the scenarios of use, values and attributes of two machine learning models for predicting diabetic ulcers and amputations. The research question was addressed through semi-structured interviews, combined with three scenarios of use components.

### 2.1 Ethics statement

This study has been ethically approved by the Ethical Committee of the Faculty of Behavioral, Management, and Social Sciences (BMS) at the University of Twente (reference number 220052). All participants provided informed consent prior to the interviews, including consent for audio recording and the use of anonymized quotations in publications. All data were anonymized prior to analysis to ensure that no individual participants could be identified.

### 2.2 Participants

For this interview study, we recruited a diverse range of stakeholders to capture a comprehensive set of perspectives on how the two ML models can be used in real-world healthcare settings. A stakeholder can be a person who has experience in the treatment, management, or technical development of diabetes care, including diabetes patients, healthcare professionals, and experts involved in system integration aspects.

Diabetes patients were recruited through convenience sampling using various methods such as a general announcement on social media, followed by referrals through personal networks. They were included if they had a diagnosis of diabetes mellitus type I or II, and an age of 18 years or older. Diabetes patients experiencing cognitive or psychological impairments were excluded.

Healthcare professionals and experts contributing to system integration aspects within diabetes care were recruited from a hospital and a podiatry center in the Netherlands through purposeful sampling. Specifically, we selected healthcare professionals involved in general diabetes care as well as those with specialized expertise in the prevention and treatment of DFUs and amputations. Additionally, we recruited experts focusing on the development, data management, and integration of technical solutions within diabetes care. All participants were required to sign the informed consent form and possess the ability to understand and speak the Dutch language to participate in the study.

Eleven stakeholders participated in the interviews, of whom 5 were diabetes patients and 6 other stakeholders, namely a physician assistant and surgical resident (healthcare professionals), data steward, technical physician, research coordinator, and a biomedical engineer (experts involved in system integration aspects). The stakeholders include potential end users (diabetes patients, healthcare professionals) and experts involved in the system's development across ethical, technical, and scientific domains, ensuring its feasibility, implementation, and practical applicability. In Table 1, the demographic characteristics of the participants are shown. No formal data saturation criterion was applied to determine sample size; the number of participants was primarily determined by practical constraints, including participant availability and the scope of the study. The sample size and composition were considered adequate given the exploratory aim of the study and the diversity of stakeholder perspectives represented.

**Table 1:** Characteristics of participants.

	*n*	Age (sd)	Female (%)	Male (%)
Diabetes patients	5	54.4 (19.0)	3 (60.0)	2 (40.0)
Other stakeholders	6	35.5 (12.3)	1 (16.7)	5 (83.3)
* System integration experts*	4			
* Healthcare workers*	2			

### 2.3 Materials and procedure

Potential participants were approached via email, which included a brief explanation of the study, an information sheet, and the consent form, inviting them to participate. Eligible participants could respond via email or phone to schedule an interview. Both in-person and online interviews were conducted based on participants' preferences. In in-person interviews, the consent form was signed on-site, whereas in online interviews, the consent form was digitally signed before the interview. The interviews with diabetes patients were carried out by a female BSc student and with the other stakeholders by a male BSc student, who both received education in conducting qualitative research. There was no prior relationship established with the participants apart from the recruitment and planning required. The interviews took place with only the interviewer and the interviewee present.

For diabetes patients and other stakeholders, a slightly different topic list was used to structure the interview based on the Unified Theory of Acceptance and Use of Technology (UTAUT) model (28). For diabetes patients, the semi-structured interview schedule comprised five topics: expected benefits, facilitating conditions, privacy, voluntary usage, and social influence. The following six topics were covered in the interviews with other stakeholders: expected benefits, facilitating conditions, privacy, social influence, hedonistic motivation, and work experience. The full interview guides are provided in the Supplementary Material (Supplementary Tables S7, S8).

At the end of the interview, three possible components of scenarios illustrating different ways in which patients can be informed about an increased risk were presented and discussed. These included the patient being informed via a mobile application, during their next appointment, or by the healthcare worker through a telephone call. Scenario components were used because the current study focuses on a technology that hasn't been developed yet, and scenario components can explicate possible options, serving as a tool to assist stakeholders in clearly articulating their needs and preferences. These scenario components were explicitly introduced as possible options, serving only to stimulate discussion rather than directly informing the scenarios of use.

### 2.4 Data analysis

As a first step, recorded interviews were transcribed using Amberscript software. The interview data was analyzed partly following the method outlined by van Velsen (32). Although the interview topic lists were structured using the UTAUT model to ensure that relevant behavioral determinants of technology acceptance were covered during data collection, the analysis itself followed an inductive, data-driven approach. This choice was made because the study aimed to generate novel, context-specific insights into scenarios of use and stakeholder values for a not-yet-developed technology, rather than to test or confirm acceptance according to a predefined theoretical model. An inductive approach was considered more suitable for capturing the range and nuance of stakeholder perspectives without constraining them to existing UTAUT constructs. As a first step, relevant fragments were selected that captured aspects related to the overall goal of the technology: facilitating timely interventions to prevent diabetic foot ulcers and amputations. Each fragment was analyzed to identify the corresponding attribute or attributes it reflects. An attribute serves as a concise summary of the key idea expressed by the stakeholders. Fragments that align with the same attribute were merged. Next, scenarios of use were formulated by directly linking attributes that suggested a particular way the technology could be implemented. In parallel, attributes were translated into corresponding values, which formed the foundation of the value specification process. In the final step, the scenarios of use were elaborated using the raw interview data to provide a detailed and contextualized description. The data analysis process was not entirely linear; insights from later steps occasionally informed earlier stages, allowing for refinement and deeper understanding throughout the analysis.

To ensure reliability in the coding process, 20 percent of the fragments (*n* = 49) were randomly selected for double coding by another researcher. Initial agreement between coders (SK & ItK) was 63.3% for values. Differences in the coding of values and attributes were resolved through discussion between the two coders until consensus was reached. The refined coding scheme resulting from this process was subsequently applied to the full dataset.

## 3 Results

In this section, we first present each scenario of use and the values and attributes.

### 3.1 Scenarios of use

**Figure 1:**
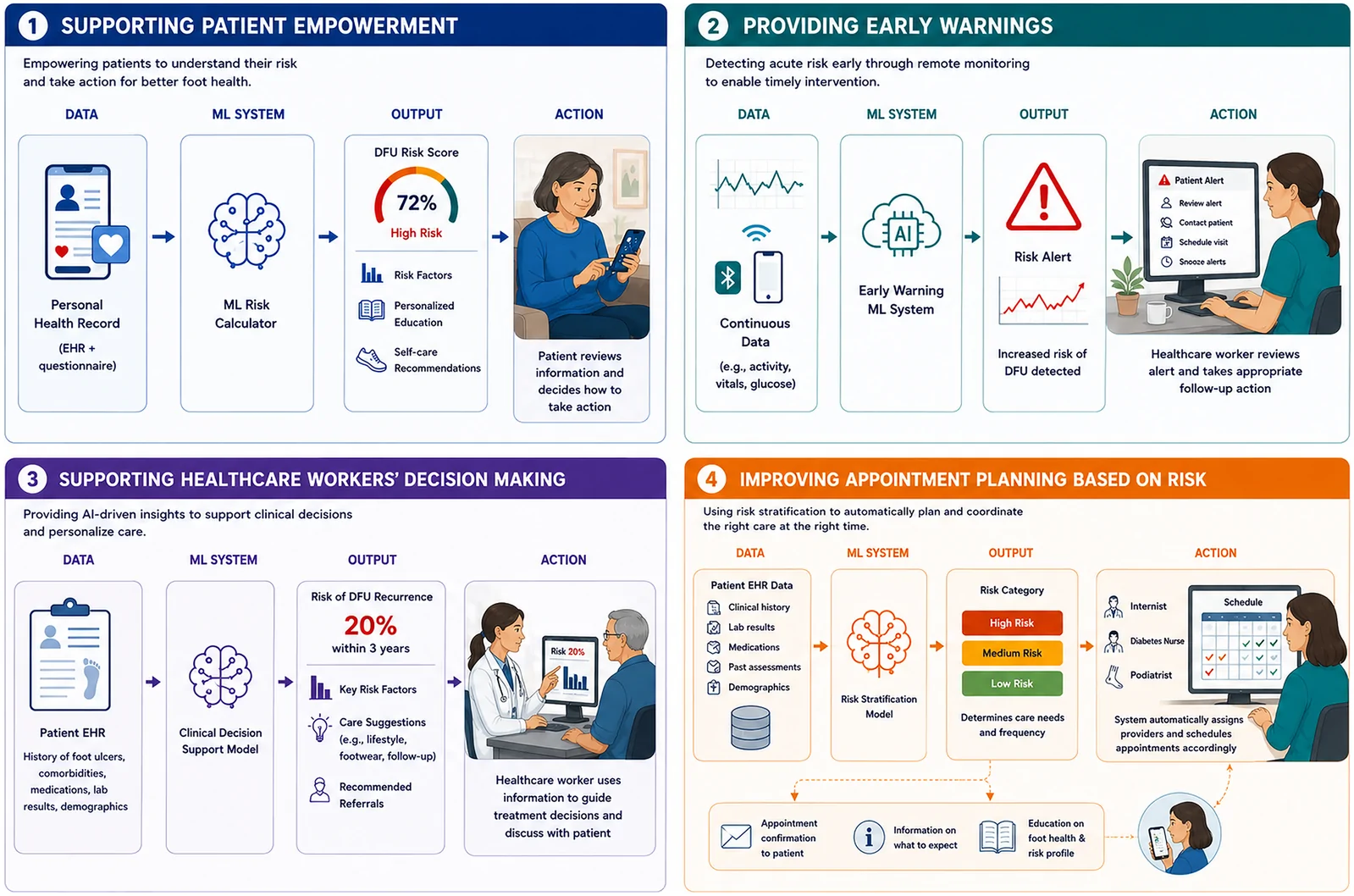
Infographic illustrating four scenarios for applying machine learning to predict DFUs and amputations: (1) patient empowerment through personalized risk scores and self-care recommendations, (2) early warning alerts for healthcare workers based on remote monitoring, (3) clinical decision support for treatment discussions, and (4) risk-based appointment scheduling.

We identified four scenarios of use for the two machine learning models through interviews with stakeholders in diabetes care: supporting patient empowerment, providing early warnings, supporting healthcare workers' decision making, and improving appointment planning based on risk (see Figure 1). The first scenario focuses on enhancing patient autonomy by using the ML model to inform patients about their risks and providing timely education for self-management (Supplementary Table S1). The second scenario aims to provide healthcare providers with early warnings of potential complications, enabling preventive measures through real-time data, such as home self-monitoring (Supplementary Table S2). The third scenario assists healthcare providers in making informed decisions about diabetes care, utilizing data from EHRs to improve the accuracy of interventions (Supplementary Table S3). The final scenario of use optimizes care pathways by scheduling appointments based on risk levels, ensuring high-risk patients receive timely interventions and reducing unnecessary appointments for low-risk patients (Supplementary Table S4). An overview of attributes linked to scenarios of use can be found in Supplementary Table S5.

### 3.2 Values and attributes

We identified 13 values and 26 attributes through the interviews. Descriptions of all values and attributes can be found in Supplementary Table S6, and the number of times values and attributes were mentioned by participants is shown in Table 2.

**Table 2:** Identified values and attributes.

Values (# participants)	Attributes	# Quotes from healthcare workers (# Healthcare workers)	# Quotes from experts involved in system integration aspects (# Experts involved in system integration aspects)	# Quotes from diabetes patients (# Diabetes patients)
Supportive patient awareness (11)	(how to) Inform patient about the increased risk for a DFU or amputation.	3 (2)	9 (4)	11 (4)
	Informing the patient must be done in a reassuring manner.	2 (1)	7 (3)	17 (4)
	Only inform the patient when the increased risk is explained by changeable parameters.	—	2 (1)	1 (1)
	Providing patients education about how to decrease the risk for DFU and amputation	4 (2)	7 (3)	7 (3)
Hybrid approach (6)	Increased risks should be validated by a healthcare worker.	—	7 (3)	—
	Support should be tailored to patients’ health literacy and motivation.	12 (2)	2 (1)	2 (1)
Acute risks (3)	Integrate self-monitoring data to enhance early detection and personalized alarms	—	4 (2)	1 (1)
Collaboration between healthcare providers at different levels (10)	Clear role division	4 (2)	9 (4)	6 (4)
Unobtrusiveness (3)	Minimizing alarm frequency to avoid overload	1 (1)	4 (2)	—
	Require minimal daily measurements	1 (1)	—	—
Integration into existing systems (9)	Integration of the system into applications that patients already use	—	1 (1)	8 (4)
	Leverage existing workflows	1 (1)	9 (4)	–
Explainability (2)	Alarms clearly explain triggers and risk factors	—	3 (2)	—
	Show limitations of the ML algorithms	—	1 (1)	—
Clinically relevant insights (5)	Synthesizing patient data	1 (1)	9 (4)	—
	Summarize in a concise manner	—	3 (1)	—
	Provide specific recommendations	1 (1)	2 (2)	—
Risk-based stratification of care (6)	Adaptive care scaling	—	6 (2)	4 (3)
	Consider patient's existing appointment schedule	1 (1)	2 (1)	—
Continuous development (5)	Stakeholders should be involved in development iterations	3 (2)	10 (3)	—
	Expanding functionality over time	1 (1)	4 (1)	—
Interoperability (2)	Integrate patient data from various healthcare providers	—	7 (2)	—
Privacy (10)	Safe data storage	—	3 (2)	5 (4)
	Risks should only be calculated for a subgroup of diabetes patients	3 (1)	—	2 (2)
	Patient consent	2 (2)	4 (2)	6 (4)
Regulatory compliance (3)	Confirm to (changing) privacy regulations	1 (1)	3 (2)	—

Cell values indicate the number of coded quotes per attribute, with the number of contributing participants in parentheses. Empty cells indicate no coded quotes from that stakeholder group for that attribute.

#### 3.2.1 Patient-centered values

The most frequently mentioned value was **supporting patient awareness** (*n* = 11), reflecting the importance of enabling patients to better understand their health risks and feel more in control. Participants agreed that informing patients about an increased risk for DFU or amputation was important, though views differed on how to communicate this. Importantly, participants emphasized that information should be reassuring and, where possible, accompanied by actionable guidance. As one participant noted: “if I know that in advance, then I can still do something to adjust it. Like changing my diet or whatever. All things I could potentially still modify now.” (Participant #9, diabetes patient).

Closely related was the value of a **hybrid approach** (*n* = 6), combining digital tools with face-to-face interaction. Participants stressed that increased risks should be validated by a healthcare worker before being communicated to patients, and that support should be tailored to patients' health literacy and motivation.

**Unobtrusiveness** (*n* = 3) was identified as important for both patients and healthcare workers. Participants described that alarm frequency should be minimized for healthcare workers to avoid overload, for instance through smart threshold settings and a “snooze” function, while patients should be required to perform only minimal daily measurements. As one healthcare worker explained, daily self-monitoring can be difficult to sustain in practice: “They already had patients measure skin temperature, but it's difficult: it's a group that's hard to get on track. And then if they also have to measure the temperature of their feet every day, well, at some point that just doesn't work anymore” (Participant #1, healthcare worker).

#### 3.2.2 System and clinical values

**Integration into existing systems** was mentioned by 9 participants, emphasizing that the system should fit seamlessly into applications and workflows that patients and healthcare workers already use, such as glucose monitoring apps and EHRs, to avoid disrupting established care pathways. As one patient explained, they would prefer risk information to appear within an application they already use daily, “that I would not have to open yet another separate app to see what is in it” (Participant #7, diabetes patient).

**Collaboration between healthcare providers** at different levels (*n* = 10) emerged as a central value. Participants highlighted the need for a clear role division in responding to system-generated alerts, with one participant advocating for “one central person responsible for these notifications” (Participant #2, expert involved in system integration aspects). The role of the general practitioner was seen as important, though concerns were raised about capacity.

**Explainability** (*n* = 2) and **clinically relevant insights** (*n* = 5) were closely linked. Participants indicated that alarms should clearly explain their triggers and underlying risk factors, and that the system's limitations should be transparent to prevent over-reliance. As one participant put it: “the alarm should clearly indicate why there is an alarm … and which factors were also taken into account” (Participant #5, expert involved in system integration aspects). Beyond individual alerts, the system should synthesize patient data into actionable insights and provide specific recommendations to support clinical decision-making. As one participant described: “With AI, you can detect patterns and connections that you wouldn't see yourself because you can process large datasets over time. It provides additional possibilities and insights” (Participant #4, expert involved in system integration aspects).

**Risk-based stratification of care** (*n* = 6) was described as a key value, where appointment frequency and care intensity are dynamically adjust based on patient risk levels, ensuring high-risk patients receive timely attention while reducing unnecessary visits for low-risk patients. One participant illustrated this with the challenge of identifying care-avoidant patients: “You really need to get under the skin of those patients, I think that's also a bit of an art, and something very personal … there are quite a few patients with a wound who simply avoid care and don't come in” (Participant #5, expert involved in system integration aspects).

**Detecting acute risks** (*n* = 3) was identified as a distinct value, focusing on the timely identification of urgent DFU risks by integrating self-monitoring data such as foot temperature sensors and mobile imaging into existing systems.

#### 3.2.3 Governance and development values

**Privacy** (*n* = 8) and **regulatory compliance** (*n* = 3) emerged as foundational values. Participants stressed the importance of safe data storage, patient consent, and limiting risk calculations to a clinically defined subgroup of diabetes patients. Compliance with regulations such as GDPR and Medical Device Regulations was seen as essential, with participants emphasizing the importance of addressing these requirements early in development to avoid prohibitive costs later.

**Interoperability** (*n* = 2) was raised in the context of integrating patient data across multiple healthcare providers, which is particularly relevant given the multidisciplinary nature of diabetes care. As one participant reflected: “that multidisciplinary aspect around the diabetic foot should not be overlooked … bringing those worlds together can mean tremendous added value” (Participant #6, expert involved in system integration aspects).

Finally, **continuous development** (*n* = 5) was identified as important, with participants emphasizing the need for iterative stakeholder involvement, including nurses, legal professionals, IT departments, and insurers, and a stepwise expansion of system functionality over time.

## 4 Discussion

Four scenarios of use for the two machine learning models predicting DFU and amputations within diabetes care were identified in this study. First, ML can be used to support patient empowerment by enhancing autonomy by giving diabetes patients insight into their risk levels and providing education and resources to decrease this risk. According to literature, this can be achieved through eHealth technologies, which facilitate patient access to personalized health information and self-management tools (33). Second, the results indicated that ML can support healthcare workers by providing early warnings of acute risks, enabling timely interventions before complications arise. Third, stakeholders described that predictive ML models based on data from EHRs offer the potential for more informed clinical judgments by supporting healthcare workers' decision-making. Previous research suggests that decision support leads to enhanced clinical satisfaction and increased efficiency (34, 35). Lastly, ML can be used to improve appointment planning by aligning the frequency of appointments to the risk levels and by assigning the patient to the right healthcare worker by gaining insight into the risk factors that explain an increased risk. By monitoring patient data from EHRs, this approach may ensure that high-risk patients receive timely care while minimizing unnecessary visits, providing opportunities for improving the overall efficiency of care pathways. These findings highlight the numerous potential applications of predictive machine learning in diabetes care, provided that stakeholders are actively involved in the development process, systems are interoperable, and compliance with regulations.

Previous research in diabetes-related eHealth and interactive health technologies, such as within HCI, ubiquitous computing, and digital health intervention domains, has highlighted values such as patient empowerment, increased awareness, privacy protection, and hybrid care approaches. For instance, McGloin et al. (36) show that telemonitoring interventions grounded in empowerment approaches significantly improved patients' glycemic control and awareness in type 2 diabetes management. Similarly, Schimmer et al. (37) highlight that person-centered eHealth services for type 2 diabetes self-management are most successful when they actively reshape care roles. Patients are supported to take greater responsibility and independence in their day-to-day disease management, for example by using self-monitoring tools, while healthcare professionals remain involved to provide tailored feedback and clinical validation. This finding is particularly relevant for our study, as it mirrors the tension we observed in the scenarios of use: while ML applications can empower patients through risk awareness and education, clinical oversight remains important for interpreting predictions and guiding interventions. This aligns with findings from previous studies, which stated that integrating AI with human expertise enhances trust and effectiveness in healthcare decision-making (38, 39).

Our findings resonate with these established values but also extend them in several ways. The focus on predictive ML models for DFUs and amputations situates the discussion in a novel clinical domain, beyond glucose monitoring technologies, bringing forward distinct challenges and contextual implications. Moreover, while previous literature often treats values like empowerment or explainability across generic diabetes tools or AI in healthcare (40), our study shows how these values differentiated per scenario of use. For example, the need for unobtrusiveness to prevent alert fatigue in early warning systems, vs. the requirement for explainability in decision support scenarios. These variations underscore the importance of developing the ML model in line with the specific needs of each scenario of use and underscores the need for involving stakeholders in early stages of the development of machine learning applications (41). Thus, the added value of the ML models should not be limited to performance metrics such as accuracy and precision, but instead both technical performance and the actual impact on clinical practice and patient care should be considered (42). While performance metrics offer insights into how well the model performs on the unseen test data, they often overlook how the model can deliver value in healthcare settings, and do not provide insight into how the ML model can be implemented in healthcare applications. The values associated with different scenarios of use can vary, emphasizing the importance of a structured development process rather than a one-size-fits-all approach to ML implementation.

In contrast, our study also showed that several values emerged consistently across all scenarios of use, underscoring their central importance for the adoption of predictive ML tools in diabetes care. In particular, regulatory and privacy compliance emerged as a core value shaping both design and implementation. Previous research also pointed out that these regulations such as the European AI Act, General Data Protection Regulation, and the Medical Device Regulations might form a barrier for adoption of AI applications in healthcare (43–45), for example, due to the time and financial resources required to comply with these regulations. In the current study, participants emphasized the importance of considering these aspects early in development to avoid high compliance costs later on, which could hinder adoption. One participant cited an example where an already implemented ML technology was discontinued due to these financial burdens. This finding aligns with recent reviews on eHealth implementation, which stress that legal and ethical requirements such as GDPR, AI Act, and MDR should be addressed from the outset to enable sustainable adoption and prevent costly re-design later in the process (46, 47). Thus, proactively addressing these challenges requires integrating regulatory compliance into early development stages. Approaches such as Privacy by Design (PbD) help identify risks, embed privacy considerations, and ensure compliance early on (48).

### 4.1 Future work

The values and attributes identified do not provide a blueprint for how the system should be designed (32). Requirements need to be specified, and more systematic development is important, which can be facilitated through multidisciplinary approaches such as the CeHRes Roadmap (31, 41). Involving different types of stakeholders can help move beyond the sole focus on technological novelty (40) and instead considering the varying attitudes of stakeholder groups might enhance its acceptance (49). The formulation of values and attributes in the current study can guide the definition of requirements that serve as the foundation of an initial prototype for gathering feedback and insights from stakeholders. This feedback will be invaluable for refining and improving the system in its early stages of development.

Moreover, although stakeholders have expressed a generally positive attitude toward the potential applications of the machine learning algorithms, it is important to critically assess its added value to current practices as well as in relation to alternative solutions. For example, improving appointment planning might also be based on prediction of no-shows using predictors such as age, previous visits, weather and previous no-shows (50), offering alternative approaches to optimizing healthcare delivery. Often, these comparative assessments are carried out too late in the process, while it is essential to conduct them early to ensure the technology can be effectively implemented and sustained in a healthcare setting, helping navigate the two “valleys of death” that frequently hinder the development and adoption of new healthcare technologies (51). Early Health Technology Assessment (HTA) offers a structured framework to evaluate the added value of ML algorithm applications early on across various dimensions (52, 53), including economic or clinical outcomes depending on the application. Furthermore, the values identified in earlier stages, such as patient awareness, collaboration, and explainability, can serve as guiding principles throughout the development and evaluation process. These values should be revisited to assess whether they are realized in practice once the algorithms are implemented, ensuring that the technology aligns with the intended outcomes.

### 4.2 Strengths and limitations

In the current study, we intentionally included a diverse range of stakeholders, namely healthcare workers, stakeholders involved in system integration aspects and diabetes patients, to capture multiple perspectives on the use of two ML models in diabetes care. This diversity is a strength, as it provides a rich starting point for understanding how the two ML models can be implemented in practice. The study involved a small sample size of eleven participants, which aligns with the exploratory nature of qualitative research aimed at gaining in-depth insights rather than achieving generalizability. However, there may have been self-selection bias, as individuals who chose to participate might have more positive attitutes towards the use of ML models in diabetes care or more motivated to contribute to solutions to healthcare challenges. As a result, the findings may reflect more optimistic perspectives. Moreover, perspectives from broader stakeholders, such as policymakers and insurers, were not included in this study. Their involvement becomes more relevant in later stages of adoption and scaling, where considerations such as upscaling, reimbursement strategies, and alignment with healthcare policies play an important role. Another point for discussion is the potential influence of the scenario components presented during the interviews. While this approach served as a tool to clearly articulating needs and preferences, these scenario components may still have guided participants' responses by framing certain possibilities or drawing attention to specific aspects of the ML model's implementation.

## 5 Conclusion

This study explored the scenarios of use, values and attributes of two ML models predicting DFUs and amputations in healthcare, contributing to a better alignment between technology, context and stakeholder needs, which facilitates successful implementation. The four scenarios of use identified through stakeholder interviews, namely supporting patient empowerment, providing early warnings, improving clinical decision-making, and improving appointment planning, not only reflect varied applications but also lead to different values and attributes. These differences underscore the importance of aligning the development of ML models to the specific needs of each scenario of use. The identified values and attributes should be regarded as preliminary design considerations rather than validated system requirements, given the exploratory nature and limited sample size of this study. They nonetheless offer an initial indication of how ML models could add value in diabetes care, for instance by supporting patient awareness, facilitating collaboration between healthcare providers, and integrating into existing systems, as well as considerations such as clinically relevant insights, interoperability, continuous development, and privacy and regulatory compliance that should be further examined in future research.

## Data Availability

The raw data supporting the conclusions of this article will be made available by the authors, without undue reservation.
