# Supplementary material for: Stakeholder perspectives on machine learning models predicting diabetic foot ulcers and amputations in diabetes care: a scenario-based interview study
Source: Front Digit Health. 2026 Aug 21;8:1773356. doi: 10.3389/fdgth.2026.1773356 (PMC13541728; doi:10.3389/fdgth.2026.1773356)
Supplement: Supplementary file 1 [file Table1.docx]

Supplementary Material

# 1 Supplementary Figures and Tables

## 1.1 Supplementary Tables

**Table S1. Scenario of use Supporting Patient Empowerment.** Rationale, PACT analysis, and example scenario illustrating the use of ML for patient empowerment.

| Rationale | Enhancing autonomy of individuals with diabetes in managing their health |
| --- | --- |
| PACT analysis | **People** – Individuals with diabetes who have various levels of eHealth and health literacy.  **Activities** – Utilizing the system to assess their risk of developing diabetic foot ulcers, understanding the contributing factors to their risk level, and receiving explanations on how to modify risk factors. Additionally, they can explore various educational topics, such as information about diabetic foot conditions and self-care practices.  **Context** – The mobile application can be accessed throughout the day and in different settings such as at home or in other daily environments  **Technology** – An add-on feature into an existing application that individuals with diabetes are already familiar with and actively using. |
| Scenario | Vera was diagnosed with diabetes 12 years ago and has been seeing an internist twice a year, once in person and once via phone, for the past three years. She also receives nurse support, sometimes remotely, depending on her schedule and health. She regularly checks her personal health record for lab results and provider notes. Within the PHR, she consented to use the ‘Personal Risk Calculator’ to assess her risk of DFUs and amputations based on her health data. After providing consent, she answered questions on health literacy and motivation to tailor support if needed. During a phone consultation, Vera was advised to wear shoes at home to prevent diabetic foot complications. However, she felt her risk was low and preferred to focus on managing glucose levels and daily activities. Occasionally, she checks the PHR, where she recently saw she was at high risk for a DFU. Clicking on the risk assessment, she learned it was validated by a healthcare provider and that preventive measures could lower her risk. Reassured but motivated, she explored the factors contributing to her risk and the educational support within the PHR to take action. |

**Table S2. Scenario of use Provide Early Warnings.** Rationale, PACT analysis and example scenario illustrating the use of ML for providing early warnings.

| Rationale | Provide early warnings when there is an acute risk of developing a diabetic foot ulcer |
| --- | --- |
| PACT analysis | **People** – Diabetes nurse and diabetes patients  **Activities** – self-monitoring by diabetes patients and reacting to warnings by healthcare workers  **Context** - The system is used in a clinical setting where healthcare professionals monitor patients remotely. Patients provide data via self-monitoring devices from their home environment.  **Technology** – self-monitoring equipment and a portal that transfers these monitoring data to EHRs |
| Scenario | A diabetes nurse, Lisa, is responsible for monitoring patients with diabetes to prevent complications such as diabetic foot ulcers. She uses the Early Warning System integrated into the hospital’s patient management system to track real-time data provided by patients through self-monitoring devices and data retrieved from electronic health records. One of her patients, John, regularly provides sensor data via a mobile app. The system detects differences in gait through the sensors, which can indicate the presence of neuropathy. An alert is generated and presented to Lisa in the dashboard, prompting her to review John's recent data. Lisa assesses the alert and decides to schedule an in-person consultation with John. Since they will have multiple contact moments over the coming weeks to address his treatment plan, Lisa utilizes the snooze button feature to temporarily disable additional alerts for the next month. This prevents redundant notifications while ensuring that John’s condition remains actively monitored through scheduled visits. After the consultation period, the snooze period ends, and the system resumes monitoring John’s data, ensuring timely alerts if new risks are detected. This feature helps Lisa manage her workload efficiently while maintaining proactive patient care. |

**Table S3. Scenario of use Supporting Healthcare Workers’ Decision Making.** Rationale, PACT analysis and example scenario illustrating the use of ML for supporting healthcare workers’ decision making.

| Rationale | Assisting healthcare workers in making better decisions about care pathways |
| --- | --- |
| PACT analysis | **People** – Healthcare workers in diabetes care, including doctors, nurse practitioners, and medical trainees, who may have limited experience or are still in education.  **Activities** - During an appointment, healthcare workers assess the patient's condition, make decisions regarding potential complications (like recurring foot ulcers), and evaluate care pathways based on the patient's historical health data and risk scores.  **Context** – During regular patient check-ups in a hospital setting, specifically when dealing with patients with diabetes who are at risk of recurring complications like foot ulcers  **Technology** – The system is integrated with the EHR, providing information about a patient's condition, risk factors, and history. It presents risk scores based on accumulated data, such as foot ulcer history, and cardiovascular complications, to assist healthcare workers in decision-making. The system also provides suggestions about necessary interventions or referrals, with the information presented in an easily accessible format within the EHR system. |
| Scenario | During a routine appointment with a patient who has a history of diabetic foot ulcers, Sophie, a medical trainee specializing in vascular surgery, opens the EHR to review the patient's past medical records. The system alerts her with a risk score, indicating that the patient is at higher risk of developing foot ulcers again, based on historical data. The system explains that, based on previous incidents, the patient has a 20% chance of recurrence within the next three years. As a trainee, Sophie finds it of added value to have this information readily available, as it helps her assess the severity of the situation. She can also see suggestions about lifestyle adjustments and recommendations for follow-up appointments. If the risk score indicates a higher risk, the system flags it, prompting her to consider early interventions or discuss referral options. However, if the risk is lower, the system suggests maintaining regular monitoring. In cases where the system generates alerts for severe risks, Sophie can address the situation promptly while ensuring that she does not feel overwhelmed by excessive information. For more moderate risks, the system’s alerts serve as a valuable guide, providing actionable insights without cognitive overload. She can use this information to make more informed decisions and discuss the findings with the patient. For Sophie, having the system highlight the most relevant patient data is particularly helpful, as it allows her to focus on patient care without the fear of missing important details. The decision support tool enhances her learning process and ensures she can provide well-informed recommendations while gaining valuable clinical experience. |

**Table S4. Scenario of use Improve Appointment Planning Based on Risk.** Rationale, PACT analysis and example scenario illustrating the use of ML for improving appointment planning based on risk.

| Rationale | Increase care efficiency by proactively monitoring risks allowing patients to receive care faster when necessary and reducing unnecessary appointments |
| --- | --- |
| PACT analysis | **People** – The care coordinator responsible for scheduling appointments, in collaboration with healthcare providers such as internists, diabetes nurses, and podiatrists.  **Activities** – Automatically scheduling follow-up appointments, assigning the right healthcare providers based on the patient's risk level, and communicating the appointments to both the patient and the healthcare team.  **Context** – A hospital setting with an integrated system for risk management and scheduling.  **Technology** - An automated system that calculates the patient’s risk level and based on that, determines the required care and appointment frequency. |
| Scenario | Sophie is the care coordinator working with an automated system to schedule appointments. Every day, she receives a new list of patients requiring follow-up appointments. The system calculates, based on their EHR data, the risk level for conditions such as diabetic foot ulcers in diabetes patients. Today, Sophie receives an alert about Vera, a diabetes patient. The system has analyzed that Vera is at high risk for developing foot ulcers due to her reduced circulation in her feet. Based on this risk level, the system automatically determines the frequency of Vera’s appointments, and the healthcare providers involved. For Vera, this means she will not only have an appointment with her internist but also with a diabetes nurse who will guide her in managing her foot care, and with a podiatrist for preventive care. The system schedules these appointments for the coming months, with shorter intervals for the diabetes nurse and podiatrist to intensify the care, given her high risk. Sophie only needs to check if the proposed appointments fit within the available timeslots of the healthcare providers. The system takes their schedules into account and automatically makes the bookings, ensuring the correct coordination of providers and timing. Vera receives an automatic appointment confirmation with all the necessary information, including what to expect from the healthcare providers and the education regarding her risk profile. |

**Table S5. Attributes linked to scenarios of use.** An x indicates that the attribute is related to the respective scenario.

| **Attribute** | **Scenario of use 1: Supporting Patient Empowerment** | **Scenario of use 2: Provide Early Warnings to Healthcare Workers** | **Scenario of use 3: Supporting Healthcare Workers’ Decision Making** | **Scenario of use 4: Improve Appointment Planning Based on Risk** |
| --- | --- | --- | --- | --- |
| (how to) Inform patient about the increased risk for a DFU or amputation. | x | x | x | x |
| Informing the patient must be done in a reassuring manner. | x | x | x |  |
| Only inform the patient when the increased risk is explained by changeable parameters. | x |  |  |  |
| Providing patients education about how to decrease the risk for DFU and amputation | x | x | x |  |
| Increased risks should be validated by a healthcare worker. | x |  |  |  |
| Support should be tailored to patients’ health literacy and motivation. | x | x | x | x |
| Integrate self-monitoring data to enhance early detection and personalized alarms |  | x |  |  |
| Clear role division | x | x | x | x |
| Minimizing alarm frequency to avoid overload |  | x |  |  |
| Require minimal daily measurements |  | x |  |  |
| Integration of the system into applications that patients already use | x |  |  |  |
| Leverage existing workflows |  | x | x |  |
| Alarms clearly explain triggers and risk factors |  | x | x |  |
| Show limitations of the ML algorithms |  | x | x |  |
| Synthesizing patient data |  | x | x |  |
| Summarize in a concise manner |  | x | x |  |
| Provide specific recommendations |  | x | x |  |
| Adaptive care scaling |  |  |  | x |
| Consider patient’s existing appointment schedule |  |  |  | x |
| Stakeholders should be involved in development iterations | x | x | x | x |
| Expanding functionality over time | x | x | x | x |
| Integrate patient data from various healthcare providers | x | x | x | x |
| Safe data storage | x | x | x | x |
| Risks should only be calculated for a subgroup of diabetes patients | x | x | x | x |
| Patient consent | x | x |  | x |
| Confirm to (changing) privacy regulations | x | x | x | x |

**Table S6. Overview of values identified along with their descriptions and an illustrative quote.** The left column lists the values, the second column provides descriptions of what each value entails, and the third column provides an illustrative quote for the value.

| **Values** | **Description** | **Quotes** |
| --- | --- | --- |
| Supportive patient awareness | Enabling patients to better understand their health risks for DFUs and amputations and feel more in control of their health through understandable risk information and educational materials. | “[…] But if I am told that I am at risk and they can’t do anything about it, well, then I’d rather not have known.” (Participant #10, diabetes patient). |
| Hybrid approach | The ML models estimate risks based on available data, but healthcare workers also play an active role in verifying and contextualizing these predictions. This collaborative process ensures that model predictions are considered alongside clinical expertise. | “I think you first need to assess it to determine how significant the risk actually is, either by nurses, a doctor, or a combination of both. Based on that, you can decide whether to inform the patient or not.” (Participant #2, expert involved in system integration aspects) |
| Acute risks | Timely identification of urgent DFU risks by integrating self-monitoring tools, such as temperature sensors and mobile imaging. | "If you could use mobile cameras or infrared technology to detect foot ulcers or areas of poor blood circulation, it might lead to earlier detection, making the alarms more timely and potentially more urgent." (Participant #4, expert involved in system integration aspects) |
| Collaboration between healthcare providers at different levels | A well-defined role division ensures efficient management of risk notifications. Having a central figure or team responsible for responding to alerts improves coordination. The involvement of general practitioners and other professionals should be balanced against their existing responsibilities. | "I always advocate for having one central person responsible for these notifications, like care activities and so on. This allows the treating physician to receive alerts and communicate directly with these patients." (Participant #2, expert involved in system integration aspects) |
| Unobtrusiveness | Minimizing alert fatigue to prevent healthcare workers’ overload by smart threshold settings and features like a ‘snooze’ function to reduce unnecessary alarms. The system should require minimal patient effort while still providing meaningful insights. | "You should not receive an alarm at the moment a patient is already under care. That is only inconvenient, and certainly for a patient. Yes, yes, actually I would also say that patients who have already had one or two wounds, well, they already have a very high chance of developing a wound. So there, you certainly should not apply the alarm. So there needs to be a good snooze option." (Participant #5, expert involved in system integration aspects) |
| Integration into existing systems | Predictive models should seamlessly fit into current digital health systems, such as glucose monitoring apps and electronic health records. Ensuring compatibility reduces additional workload for healthcare professionals and diabetes patients. | "What I would find convenient is if it could be linked to an existing system that is already there. And by that I mean: the system that I am currently wearing myself, for example. The information that I can find, the amount of insulin that I have injected, that that would also be visible in the app. That I would not have to open yet another separate app to see what is in it." (Participant #7, diabetes patient) |
| Explainability | Risk notifications must clearly convey why an alert was triggered and the underlying risk factors. Providing transparency about the ML model’s limitations prevents over-reliance on algorithmic outputs. | "But I also think that the alarm should clearly indicate why there is an alarm. Yes, and what, what event triggered the alarm, and which factors were also taken into account, that you receive a small report on why those risk factors caused the threshold to be reached." (Participant #5, expert involved in system integration aspects) |
| Clinically relevant insights | Raw data must be structured into actionable insights that support clinical decision-making. The system should highlight patterns, deviations, and risk factors in an intuitive manner. Summarized recommendations help healthcare workers quickly assess patient needs. | “With AI, you can detect patterns and connections that you wouldn’t see yourself because you can process large datasets over time. It provides additional possibilities and insights.” (Participant #4, expert involved in system integration aspects) |
| Risk-based stratification of care | By dynamically adjusting care based on risk levels, healthcare resources can be allocated more effectively. High-risk patients receive timely attention, while low-risk individuals avoid unnecessary visits. The approach must align with existing care pathways to maintain workflow efficiency. | "So a good calibration of the system, with a very good threshold of okay, when do we give such a warning and what type of warning do we give then? I think that is very important." (Participant #6, expert involved in system integration aspects) |
| Continuous development | Ongoing refinement, based on feedback from both patients and healthcare professionals, ensures the system remains aligned with stakeholders’ needs. Iterative improvements can help in adapting to the changes and allow for providing continuous feedback. | “That is the dealbreaker now; if you are not efficient with your system or it only costs money, then it's a no-go for implementing something like that. So, you really need to sit down with the stakeholders to determine where the benefit lies, and that benefit can also be quality. […]” (Participant #6, expert involved in system integration aspects) |
| Interoperability | Data exchange between different healthcare providers ensures continuity of care and informed decision-making. | “That multidisciplinary aspect around the diabetic foot should not be overlooked, so each person has their own knowledge and expertise, and bringing those worlds together can mean tremendous added value.” (Participant #6, expert involved in system integration aspects). |
| Privacy | Data protection measures are important for protection patient information. Patients should have control over how their data is used. | "Well, I assume that the government handles my data carefully. And that could be the case, it is always a possibility. I would not worry too much about it." (Participant #9, diabetes patient) |
| Regulatory compliance | Ensuring compliance to medical regulations and data protection laws is important for responsible deployment. | “[…] in the Netherlands you are dealing with very strict medical device regulations, which also apply to apps, algorithms, and so on. You then need to have a CE marking, all kinds of tests need to be conducted, et cetera. Implementing such a system or app on a structural basis also takes a lot of time and money to get properly approved." (Participant #4, expert involved in system integration aspects) |
